# Anxiety, Compassion and Pride. How Emotions Elicited by the Government’s Handling of Covid-19 Influences Health-Promoting Behavior

**DOI:** 10.5334/pb.1053

**Published:** 2021-07-23

**Authors:** Emma A. Renström, Hanna Bäck

**Affiliations:** 1 Department of psychology, University of Gothenburg, SE; 2 Department of political science, Lund University, SE

**Keywords:** Satisfaction, Emotions, Compliance, Covid-19, Sweden

## Abstract

This article explores how emotions connected to the Government’s handling of the Covid-19 outbreak in Sweden relates to behaviors to stop the spread of the virus, and which emotions functions as mediators in this relationship. The Swedish approach to handling the outbreak greatly differed from how many other Western European countries handled the situation and thus makes an important case to study. In a large representative survey (N = 2449), we found that satisfaction with how the Government handled the situation was related to more positive and less negative emotions. Anxiety, compassion and pride mediated the effect of satisfaction on compliance with the national recommendations such that anxiety and compassion increased compliance, while pride decreased it. Importantly though, satisfaction increased compassion and pride, but only compassion led to more compliant behaviors. In fact, satisfaction was indirectly related to less compliant behaviors via anxiety and pride. Shame mediated the effect on the tendency to wear face masks, a behavior that was explicitly not endorsed by the Swedish Public Health Agency. We speculate if the face mask, which was intensely debated, became a politicized symbol of dissatisfaction with the Swedish approach. In sum, it seems that individuals who were dissatisfied with how the government handled the Covid-19 outbreak were in fact engaging more in health-promotional behaviors to stop the spread of the virus.

## Introduction

The year 2020 has gone down in history as one of the most strained years ever due to the Covid-19 pandemic. It has shaken societies across the world and left none unaffected. Governments all over the world have adopted different approaches to stop the spread of the virus. Among the Western European countries, complete lockdowns were the most common strategy during the spring of 2020 (Bol et al., 2020). Ten EU members had lockdowns in place by the end of March, 2020, and in May, 2020, 161 countries worldwide had adopted lockdowns (Cheng et al., 2020). Most lockdowns entailed forced, and strictly enforced, nationwide, social confinement. In contrast to most other countries, Sweden took a different approach and left its society relatively open. Even though many were sceptic of this approach, the curve of active covid-19 cases flattened after the peak in April, 2020 (see Folkhälsomyndigheten.se for updated statistics), and cell phone data showed that the Swedes did in fact refrain from travelling during Easter (e.g. SVT, 2020).

While the Swedish approach, that essentially builds on an idea that individuals sacrifice their own freedom for the greater good of society, was celebrated by many, others were skeptical. The Swedish approach divided Swedish society, leading to polarized attitudes about how the situation was handled. However, it is unclear if this polarization influenced compliance with the recommendations made by the Swedish Public Health Agency. In this paper, we explore how satisfaction with the government’s measures to stop the covid-19 spread influenced compliance with national recommendations and other health related behaviors. Specifically, we explore the mediating role of emotions elicited by the national approach, and ask, did emotions elicited by the government’s handling of Covid-19 influence individuals’ health-promoting behavior?

### Satisfaction and compliance with restrictions

The most important goal nationwide during the Covid-19 pandemic has been to decrease the spread of the virus. To this end, severe restrictions on individual freedom were endorsed. To understand what influence individuals’ compliance with such restrictions is therefore a key question. To answer this question, we can draw on previous research on satisfaction, trust and compliance.

The way decisions are made has been shown to have important consequences for individuals’ willingness to follow the decisions. For instance, the procedural justice literature shows that when people are satisfied with the way a decision has been reached, they tend to obey to the outcome even if they preferred another alternative (Bäck et al., 2011; Grimes, 2006; Tyler, 2015). This effect has also been found when it comes to how politicians make decisions that may be unfavorable to citizens (Esaiasson, 2010). People also become more accepting and less polarizing among their attitude allies and opponents when procedures are seen as sound (Bäck et al., 2010).

Similarly, trust in authorities also show that people tend to follow the recommendations put forth by these authorities (Prati et al., 2011; Levi, 1996). During the initial phase of the Covid-19 pandemic, many citizens’ levels of trust in their authorities increased (Sibley, et al., 2020), which also happened in Sweden (Esaiasson et al., 2020). As more studies are coming out, many of them focus on trust as a predictor for compliance with national guidelines and lockdowns, but fewer seem to focus on satisfaction with how the authorities handled the outbreak. Although, confidence in how the government has tackled the pandemic was strongly related to intent to get vaccinated, according to a recent Belgian report (Motivation Barometer, 2021), indicating that perceptions of how the outbreak was handled by authorities matters for health related behaviors.

Leadership is another important aspect in compliance. Especially, leaders that can convey a shared social identity among the public – that “we are all in this together”, seem to be successful in promoting compliance (Haslam et al., 2011; Fransen et al., 2015). This was in many ways particularly true in Sweden, where the head of the Swedish Public Health Agency became the face of the architecture of the Swedish approach. Importantly, satisfaction has been shown to be related to compliance in a variety of settings (Albrecht & Hoogstraten, 2007; Esaiasson, Dahlberg & Kokkonen, 2020).

Hence, we expect that satisfaction with how the Government and the Public Health Agency handled the Covid-19 outbreak will affect level of compliance with national recommendations and other health related behaviors to stop the spread of the virus. However, this is not necessarily the case for the Covid-19 situation, since people who were dissatisfied with the handling of the situation may still be prone to follow the recommendations and engage in other health promotional behaviors to limit the spread of the virus regardless of their level of satisfaction. Thus, to better understand the motivation of health-promotional behaviors, we turn to emotions as mediators.

### Emotions as mediators

Satisfaction with procedures is connected to emotions (Hegtvedt & Killian, 1999; Krehbiel & Cropanzano, 2000) and from the public debate, it is obvious that emotions surrounding the Swedish approach are strong and varied. Emotions have a long-standing history in psychology and the way they have been defined has varied over the centuries. Contemporary psychology identifies some features of emotions that separate them from other constructs, such as mood or feelings. For instance, emotions relate the self to an object through appraisals of that object (Arnold & Gasson, 1954). Frijda and Mesquita (1994) for instance, claim that emotions are “modes of relating to the environment: states of readiness for engaging, or not engaging, in interaction with that environment”. This means that emotion episodes typically have an object (Frijda, 1993). The object is then evaluated through an appraisal process (Arnold & Gasson, 1954; Lazarus, 1991). According to Lazarus (1991) an emotion is a response to an evaluative judgment.

In relation to the Covid-19 outbreak, the appraisal of how the situation was handled by the authorities may therefore have led to different emotional reactions. For instance, those who were satisfied with the handling should feel positive emotions, while those unhappy with the handling should feel more negative emotions.

One reason that emotions are important to human life is that they are likely to influence action tendencies. The emotional reaction informs an individual about a situation and subsequently prepare the body for a certain course of action (Frijda, 1986). Emotions hence influence behavior, such as the basic fighting or fleeing, but emotions also influence cognitive processing, which may become either more or less elaborate, depending on which emotions are elicited by a certain stimulus (Izard, 2010). In the present article, we are interested in how emotions specifically related to the Swedish approach may influence compliance with national recommendations and other health related behaviors. A large body of research indicate the focal position of emotions in health related decision-making and behaviors (for an overview, see Ferrer & Mendes, 2018).

There are several different theories on how emotions should be conceived of ranging from a set-up of distinct emotions to a dimensional approach where different emotional states are more fluid. Regardless of how emotions are described, a broad distinction is made by valence, that is the positive or negative nature of emotions. In the past, negative emotions have often been lumped together, regardless of their specific nature, including anger, fear, anxiety, sadness and so on (see e. g. Brader & Marcus, 2013 for an overview). The widely influential PANAS is an example of this. PANAS stands for Positive Affect (PA) and Negative Affect (NA), and suggest that affect should be conceptualized in terms of these two dimensions (Watson, Clark & Tellegen, 1988).

However, much research suggests that different negative emotions have extensively disparate impact on cognition and behavior (Bodenhausen et al., 1994; Banks & Valentino, 2012; Merolla & Zechmeister, 2009; Marcus et al., 2000; Harmon-Jones et al., 2014; Harmon-Jones et al., 2009). For instance, anger has been related to approaching behavior, and to limiting cognitive processing, and reliance on heuristics and stereotypes (Carver & Harmon-Jones, 2009; Harmon-Jones, Harmon-Jones, Abramson, & Peterson, 2009; Harmon-Jones, Price, Gable, & Peterson, 2014). While fear is likely to have similar effects, given that it is accompanied by a high level of arousal (Witte, 1992; Witte, 1998), preparing for action, although rather in the form of fleeing, anxiety has other effects (Sylvers et al., 2011; Renström & Bäck, 2021). Anxiety is elicited by an uncertain threat and leads to more elaborate cognitive processing. Even though the effects are clearly different, fear and anxiety are often grouped together (Renström & Bäck, 2021).

In addition to the basic emotions anger, fear and anxiety, we here explore other negative emotions such as shame and sadness, which may influence behavior (Gausel & Brown, 2012). Moreover, positive emotions may also be important as positive emotions has been shown to influence positive health behavior change (Van Cappellen et al. 2017). Van Cappellen and colleagues (2017) present a theoretical framework where positive affect experienced during performing positive health behaviors feeds into an upward spiral which then influences everyday decisions to repeat such behaviors. In this article we explore the role of pride, which has been shown to influence behavior (Lea & Webley, 1997; Wubben et al., 2012).

Another positive emotion is compassion, which is a “strong feeling of sympathy with another person’s feelings of sorrow or distress, usually involving a desire to help or comfort that person (APA Dictionary). Based in evolutionary psychology, compassion can be seen as having been evolved from an adaptive focus on protecting oneself and one’s offspring to a broader focus on protecting others including and beyond one’s immediate kinship group (de Waal, 2009). As such, it is not surprising that compassion, or empathy, has been shown to increase compliance with restrictive behavior during the Covid-19 pandemic (Pfattheichera et al., 2020).

Hence, we here explore a wide range of emotions that people experience when thinking about the Swedish approach, and how these relate to health promoting behavior. Most importantly, we believe that some specific emotions, such as anxiety, compassion and pride will mediate the impact of satisfaction with the government’s handling of the pandemic on the individual’s likelihood of complying with government recommendations. We also explore one health promoting behavior that was not endorsed by the Public Health Agency – the face mask. Hence, we are able to determine the mediating effects of emotions from satisfaction for health promoting behaviors that both relate to compliance with recommendations, but also that were not related to compliance.

### The Swedish case – a “lenient” approach to battling Covid-19

In an overview of policies implemented in reaction to Covid-19, Cheng et al. (2020) present data of over 10 000 policy announcements in over 190 countries. They find that the most commonly implemented policy is to close national borders. The second most common policy is to close schools and non-essential businesses. In most cases, the implementations were also compulsory. Such measures undoubtedly affect citizens both individually and as a nation. A growing literature focuses on the negative health aspects of confinement (e.g. Arora & Grey, 2020).

The Swedish approach has differed compared to most other countries in that Sweden did not go for a lockdown strategy, even though the goal – to “flatten the curve” was the same in Sweden as in other countries (Ludvigsson, 2020). The reason behind this more lenient approach has been to uphold a society and keep the economy from a complete dead stop, and allow some sort of normalcy of life. Instead of the restrictions that were regulated by law enforcement, which most other countries opted for, Sweden’s Public Health Agency provided recommendations for individual behavior. The term “recommendation” is culturally dependent, and also varies depending on who makes such a recommendation. As the Swedish Prime Minister, Stefan Löfven stated in a rare speech to the public on March, 22, when the Public Health Agency gives a recommendation, it is expected that the public complies – it is not a choice to do it or not, you should do it. Still, there was no punishment for citizens if they did not follow the recommendations. The recommendations were among others to keep distance, avoid non-essential travels, increased hand hygiene, and to stay at home and work from home as much as possible.

Even though restrictions for bars and restaurants were legally enforced, individuals’ freedom was still largely unregulated. Shops and businesses remained open during the crisis during the entire spring, as did pre-schools and basic schools. Even though many businesses struggled, most people could still work, either from home or at their work place. Children could keep up relatively normal lives, and the general psychological distress should at least be lower as compared to many other countries. This relatively lenient strategy makes Sweden an excellent case for exploring how the recommendations presented by the Public Health Agency affected actual behaviors.

The case of Sweden has both shocked and amazed across the world (Miltimore, 2020). Some have watched in disbelief, but as the data came in, it seemed as if the recommendations had affected the Swedes’ behavior. For instance, data from cell phone companies showed that travels during the Easter break was severely reduced (e.g. SVT, 2020). Nonetheless, the debate about the “correct” approach is still ongoing (December, 2020) and stirring emotions both within Sweden and internationally. Early summer 2020 (June) Sweden had much higher Covid-19 mortality rates compared to, for instance, the other Nordic neighbor countries which employed lockdown strategies. The Swedish approach has been highlighted in the media and presented both as a horrific experiment with its population and as a successful approach that allows for long-term compliance with minimal damage to the economy (Holmes et al., 2020; Huang & Zhao, 2020; Li et al., 2020).

Nonetheless, it is clear that the Swedish approach put forth by the Swedish Public Health Agency and endorsed by the Swedish Government, was not supported by all Swedish citizens. In fact, several doctors and researchers has put forth skepticism. In late March, 22 researchers wrote an open letter criticizing the approach (Trysell, 2020). The Chief physician and professor of infection medicine, Björn Olson, has repeatedly criticized the State epidemiologist and head of Public Health Agency, Anders Tegnell, in media (e.g. Claesson, 2020). Even the former State Epidemiologist Annika Linde, criticized the Swedish approach in international media (Orange, 2020). But the Government supported the Public Health Agency, which also stood its ground. These cleavages rubbed off on the public. In a blog post on *The Conversation*, a survey of 1600 Swedes revealed major divisions in attitudes to the Swedish approach (Franks & Nilsson, 2020). On various discussion forums and Twitter threads, the debate between citizens was heated. One particular issue that entered the spotlight was the divide about the face masks. The Public Health Agency recommended against the use of face masks with the motivation that it would provide a false sense of security and that people then would not feel the need for physical distancing. In addition, they claimed that there was no empirically determined evidence that face masks contributed to lowering the spread of the virus. This stance greatly deviated with the rest of the Western World and again, scientists were dissatisfied with the recommendations and published a debate article arguing for why the arguments of the Public Health Agency’s recommendation against face masks were invalid (Dagens Nyheter, 2020; Rocklöv & Rootzen, 2020).

## Methods

### Design

The analyses presented here are based on an online survey with a cross-sectional, non-experimental design. The aim was to explore how satisfaction with the governmental handling of the Covid-19 outbreak influence compliance with the recommendations, and if emotions related to the Swedish strategy function as mediators in this relationship. Data was collected by the survey company Enkätfabriken, 25^th^ of August – 10^th^ of September, 2020. The survey was designed using Qualtrics. The study complies to ethical guidelines as specified by the Swedish Ethical Authority.

### Procedure

Participants were invited to take part in a survey about *Feelings and opinions in relation to the Covid-19 virus*. Participants were first informed about the study and its purpose, how data was to be treated and their right to withdraw. Before starting the survey, the participant was required to provide informed consent. First, they were asked questions about ideological position, after that followed questions about the handling of the Covid-19 outbreak. Then we assessed emotional experience in relation to the handling of the outbreak, and then we assessed health promoting behaviors. Finally, we asked some demographic questions such as age, gender and education level.

### Participants

In total, 2449 participants filled out the survey. Mean age was 48.89 (*SD* = 17.19), ranging from 18–91, which is a little bit older than average in Sweden: 41.2 (Statistics Sweden, 2021). There were 1173 (47.90%) women, 1101 (45%) men, 6 (0.24%) non-binary, 1 (0.04%) trans person and 168 (6.9 %) participants did not answer the gender question. The sample was fairly highly educated compared to average numbers in Sweden (Statistics Sweden, 2021). Three (0.12%) participants not having completed basic schooling, 115 (4.70%) having basic schooling, 738 (30.13%) having completed high school, 263 (10.73%) having completed vocational post-high school training, 1144 (46.71%) having a higher education (university/college), and 32 (1.31%) having a post-graduate degree. There were 154 (6.29%) participants who did not respond to the education question.

The sample were centrist both regarding left-right positioning and liberal-conservative positioning, *M* = 5.55 (*SD* = 2.48), and *M* = 4.80, *SD* = 2.13, on 10-point scales. Finally, the sample reported fairly high on political interest, *M* = 5.89 (*SD* = 2.61), on a 10-point scale.

### Measures

To assess *satisfaction with the handling* of the situation we asked the question *How well do you think that the government and Public Health Agency has handled the situation with the outbreak of Covid-19 when it comes to limiting the spread of the virus?* Then we assessed three items: *The efficiency of the measures taken; How fast the measures were taken*; and *The extent of the measures taken*. Answers ranged from *1 = Not well at all*, to *7 = Very well*. The items were combined into a mean index with Cronbach’s α = 0.90.

To assess discrete emotions, we adapted the Discrete emotions questionnaire (Harmon-Jones et al., 2016) to fit our purposes and we also included more emotions to capture the variations. The question read: *Sweden’s way of handling the Covid-19 outbreak has been very different from many other countries. When you think about how Sweden has handled the outbreak, what emotions do you then experience?* We then listed 31 discrete emotions in a matrix format, and participants rated for each emotion their experience on a scale from *1 = Do not experience* at all, to *7 = Powerful experience*. We then created mean indices of discrete emotions, as presented in ***Table 1***.

**Table 1:** Discrete emotions items.


INDEX	ITEMS	M (SD)	ALPHA

Anger	Anger	2.36 (1.45)	0.93

	Wrath		

	Frustration		

	Rage		

	Irritation		

	Uppsettedness		

Fear	Fear	2.12 (1.34)	0.90

	Alarm		

	Panic		

	Terror		

Anxiety	Anxiety	2.62 (1.40)	0.93

	Concern		

	Unpleasantness		

	Helplessness		

	Nervousness		

	Apprehension		

	Dread		

Sadness	Sadness	2.73 (1.51)	0.83

	Sorrow		

	Emptiness		

Shame	Shame	1.85 (1.28)	0.89

	Degradation		

	Dishonor		

Pride	Satisfaction	3.24 (1.34)	0.86

	Pride		

	Honor		

	Dignity		

	Self-respect		

	Happiness		

Compassion	Empathy	4.19 (1.54)	0.80

	Compassion		


To assess compliance with recommendations, we asked the question: Do you do anything yourself to decrease spread of the virus? In a matrix format, participants rated the items: Avoid travelling; Avoid meeting other people; Keep at least 1 meter distance to others; Wash/disinfect the hands more than usual. Each item was rated on a scale from *1 = Not at all*, to *7 = As much as I can*. Cronbach’s alpha of an index of compliance was 0.75.^1^

Finally, we assessed one type of behavior to limit the spread that was not recommended by the Public Health Agency and that was highly discussed – wearing face masks. It was assessed on the same scale as the compliance items, but used in the analyses as a single item.

We include several control variables in our analyses, for example measuring respondents’ age, gender (as free-text response), education, and self-positioning on a left-right scale *(1 = Clearly to the left, 10 = Clearly to the right)*, and a liberal-conservative scale *(1 = Clearly liberal, 10 = Clearly conservative)*, and political interest *(1 = Not at all interested, 10 = Very interested)*.

## Results

We first present descriptive results and then a parallel mediation model to evaluate whether satisfaction with the government’s handling of the covid-19 outbreak influences actual compliance with recommendations, or other health-related behaviors, depending on what emotions are evoked by the Swedish strategy.

On average, the participants were fairly satisfied with the Government’s handling of the outbreak, and there was an overall high compliance with recommendations, but the wearing of face masks was low. Compliance and satisfaction were not correlated indicating that level of satisfaction with the Government’s handling did not influence the extent to which people engaged in behaviors that aimed at limiting the spread of the virus. However, satisfaction was negatively correlated with the wearing of face masks, such that those who were dissatisfied with the handling were more prone to wear a face mask. Finally, there was a positive correlation between compliance and wearing face mask indicating that people who engage in behaviors to limit the spread do so regardless of whether the behavior is sanctioned by the Government and Public Health Agency or not. Means and standard deviations for satisfaction, compliance and wearing face mask, as well as inter-correlations are shown in ***Table 2***, while the emotion indices can be seen in ***Table 1***.

**Table 2:** Means, standard deviations and correlations for the independent and dependent variables.


	M (SD)	SATISFACTION	COMPLIANCE

Satisfaction	4.56 (1.60)		

Compliance	5.78 (1.16)	–0.004	

Face mask	1.61 (1.41)	–0.21***	0.11***


**Figure 1:**
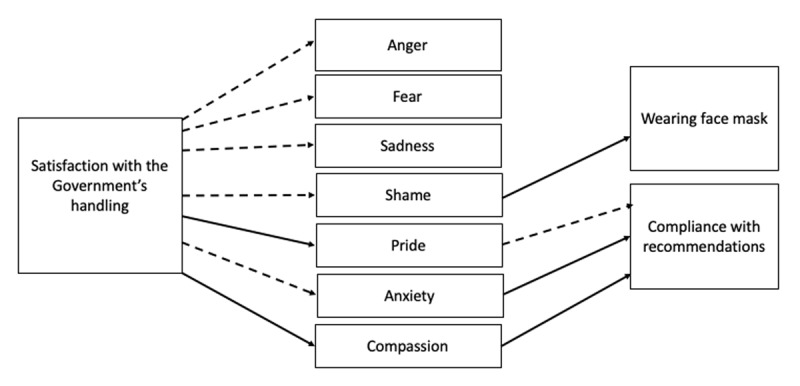
Parallel mediation models showing significant paths from satisfaction to emotion and from emotions to compliance. *Note*: Dashed paths show negative effects and solid paths show positive effects.

To explore the mediating role of emotions elicited by the Swedish approach between satisfaction with the Government’s handling and compliance with recommendations and another health related behavior, the wearing of face mask, which was not recommended by the Public Health Agency, we ran two separate parallel mediation models (model 4, Hayes, 2013). As control variables we included binary gender (man = 1, woman = 0), age, education level (used as continuous variable), self-position on a left-right scale, self-position on a liberal-conservative scale, and political interest. The independent variable was satisfaction with the Government’s handling, and as mediators we entered the emotion indices (anger, fear, sadness, shame, pride, anxiety, compassion). The outcome variables were compliance with recommendations, and wearing face mask. The results are presented in ***Tables 3*** and ***4***, and illustrated in ***Figure 1***.

**Table 3:** Parallel mediation models for compliance with national recommendations.


	ANGER (M1)	FEAR (M2)	SADNESS (M3)	SHAME (M4)	ANXIETY (M5)	COMPASSION (M6)	PRIDE (M7)	COMPLIANCE (Y1)	FACE MASK (Y2)

ANTECEDENT	COEFF	COEFF	COEFF	COEFF.	COEFF.	COEFF	COEFF.	COEFF.	COEFF.

Constant	4.58 (0.17)***	4.16 (0.18)***	4.32 (0.20)***	3.50 (0.16)***	4.58 (0.18)***	3.02 (0.21)***	1.58 (0.17)***	4.75 (0.18)***	1.15 (0.23=***

Anger (M1)	–	–	–	–	–	–	–	–0.005 (0.03)	0.06 (0.02)

Fear (M2)	–	–	–	–	–	–	–	–0.002 (0.04)	0.08 (0.05)

Sadness (M3)	–	–	–	–	–	–	–	0.02 (0.03)	0.01 (0.03)

Shame (M4)	–	–	–	–	–	–	–	–0.03 (0.03)	0.12 (0.03)***

Anxiety (M5)	–	–	–	–	–	–	–	0.13 (0.04)**	0.03 (0.05)

Compassion (M6)	–	–	–	–	–	–	–	0.07 (0.02)***	–0.001 (0.02)

Pride (M7)	–	–	–	–	–	–	–	–0.05 (0.02)*	–0.01 (0.03)

Satisfaction (X)	–0.46 (0.02)***	–0.28 (0.02)***	–0.35 (0.02)***	–0.30 (0.02)***	–0.34 (0.02)***	0.24 (0.02)***	0.038 (0.02)***	–0.001 (0.02)	–0.05 (0.02)*

Left-right (C1)	0.02 (0.01)	–0.00 (0.01)	0.00 (0.01)	0.02 (0.01)*	–0.01 (0.01)	–0.04 (0.01)*	0.01 (0.01)	–0.02 (0.01)	–0.02 (0.01)

Liberal-Cons. (C2)	0.05 (0.01)***	0.05 (0.01)***	0.03 (0.02)*	0.06 (0.01)***	0.05 (0.01)**	–0.04 (0.02)*	–0.02 (0.01)	–0.02 (0.01)	0.03 (0.02)

Age (C3)	–0.004 (0.00)*	–0.01 (0.00)***	0.01 (0.00)**	–0.01 (0.00)**	–0.005 (0.00)**	0.004 (0.00)*	–0.004 (0.00)*	0.02 (0.001)***	–0.002 (0.001)

Gender (C4)	–0.24 (0.05)***	–0.40 (0.05)***	–0.38 (0.06)***	–0.07 (0.05)	–0.39 (0.06)***	–0.31 (0.07)***	–0.06 (0.05)	–0.35 (0.05)***	–0.12 (0.06)*

Education (C5)	–0.09 (0.03)***	–0.11 (0.03)***	–0.10 (0.03)**	–0.10 (0.02)***	–0.07 (0.03)**	0.02 (0.03)	0.01 (0.03)	0.05 (0.02)*	–0.02 (0.03)

Political interest (C6)	0.04 (0.01)***	0.02 (0.01)	0.04 (0.01)**	0.03 (0.01)***	0.03 (0.01)**	0.05 (0.01)***	0.02 (0.01)*	–0.03 (0.01)**	0.04 (0.01)***

	R2 = 0.31	R2 = 0.17	R2 = 0.16	R2 = 0.22	R2 = 0.19	R2 = 0.10	R2 = 0.21	R2 = 0.13	R2 = 0.11

	F(7,2113) = 134.25***	F(7,2113) = 62.17***	F(7,2113) = 58.89***	F(7,2113) = 86.00***	F(7,2113) = 70.52***	F(7,2113) = 35.06***	F(7,2113) = 81.30***	F(14,2106) = 21.42***	F(14,2099) = 17.61***


*Note*: *** p < .001, ** p < .01, * p < .05. Gender is dummy coded with women = 0, men = 1.

**Table 4:** Direct and indirect effects of satisfaction with the Government’s handling on compliance.


	COMPLIANCE (Y1)	FACE MASK (Y2)

*Direct effect*	–0.001 (–0.04; 0.04)	–0.05 (–0.10; –0.003)

*Indirect effects*		

Anger	0.002 (–0.03; 0.03)	–0.03 (–0.07; 0.02)

Fear	0.001 (–0.02; 0.02)	–0.02 (–0.06; 0.01)

Sadness	–0.01 (–0.02; 0.01)	–0.005 (–0.03; 0.02)

Shame	0.01 (–0.01; 0.03)	–0.04 (–0.07; –0.01)

Anxiety	–0.05 (–0.08; –0.02)	–0.01 (–0.05; 0.03)

Compassion	0.02 (0.01; 0.03)	–0.003 (–0.01; 0.01)

Pride	–0.02 (–0.04; –0.004)	–0.002 (–0.02; 0.02)


*Note*: Confidence intervals in parentheses. Level of confidence for all confidence intervals is 95%. Results are based on 5000 bootstrap samples.

As can be seen in ***Table 3*** and ***Figure 1***, there were significant effects of satisfaction with the Government’s handling on all emotions. For negative emotions, the direction was negative, such that higher satisfaction was related to lower anger, fear, sadness, shame and anxiety. For the positive emotions, there were positive effects such that increased satisfaction was related to more compassion and pride.

In line with previous research (Renström, Bäck & Schmeisser, 2020), left-right political orientation showed marginal effects and hence it seems that the polarized attitudes about the handling of the Covid-19 outbreak do not follow traditional party lines. There were weak, but fairly consistent effects of liberal-conservative position where conservatism was related to stronger negative emotions, and less compassion. This also aligns with recent research that show that trust in the Public Health Agency was related to more traditionalist views (Bjereld & Demker, 2020). Age was also consistently related to the emotions such that older age was related to less anger, fear, shame, and anxiety, but also less pride. Higher age was also related to more sadness and compassion. Gender was related to most emotions, such that men (coded as 1) were less emotions in general, that is, men were less angry, afraid, sad, anxious and compassionate compared to women. There was no effect of gender on shame or pride. Higher education was related to less negative emotions, but unrelated to positive emotions. Political interest was related to stronger emotions across the board, both positive and negative.

In the final step of the model, we can see that there was no direct effect of satisfaction with handling on compliance, but that three emotions were related to compliance. Anxiety and compassion were positively related to compliance such that higher levels of anxiety and compassion were related to increased levels of compliance, while pride was negatively related to compliance. That is, higher levels of pride was actually related to lower levels of compliance.

There was a direct effect of satisfaction with handling on the tendency to wear face masks, such that people who were more dissatisfied with the Government’s handling were more likely to wear a face mask, even though the effect was weak. Interestingly, shame had a significant effect on wearing face masks, such that when people felt more shame in relation to the Swedish approach, they were also more likely to wear face masks.

***Table 4*** shows if the indirect effects were significant and as can be seen, for compliance the indirect effect of anxiety, compassion and pride were significant, and for wearing face masks, the indirect effect of shame was significant.

The results hence indicate that satisfaction with the Government’s handling, to some extent is related to compliance with the recommendations, and the tendency to wear face masks, via emotions. People who are satisfied with the handling show more compassion and pride, but only compassion is related to increased compliance, while pride actually has the opposite effect. So, feeling proud about the Swedish approach is related to less compliance with the national recommendations. More satisfaction was related to lower anxiety, but high anxiety was related to lower compliance. Hence, lower anxiety in this case is actually not beneficial for the greater good. In this way, people who are satisfied seem to be less compliant with the national recommendations. Moreover, being dissatisfied with the handling led to feeling shameful of the Swedish approach, which in turn increased the tendency to wear face masks, that is, a health promoting behavior not endorsed by the Public Health Agency.

In sum, our results indicate that overall, there seems to be more positive effects on health promoting behaviors of being dissatisfied with the Government’s handling.

## Discussion

This study sought to explore if the attitudinal divide among Swedish citizens about how the Swedish Government handled the Covid-19 outbreak was related to compliance with the national recommendations, and other behaviors to contain the spread of the virus. The results confirmed that emotions related to how the situation was handled in Sweden were highly varied. First, satisfaction with the handling was related to lower scores on all negative emotions, and higher scores on all positive emotions, as would be expected. Furthermore, satisfaction with the Government’s handling had no direct effect on compliance, but a weak effect on the tendency to wear face masks, where dissatisfaction was related to higher inclination to wear a face mask. The effect on compliance was mediated by three emotions; anxiety, compassion and pride, while wearing face masks was mediated by shame. While anxiety and compassion were related to increased compliance, pride was related to decreased compliance. Noteworthy is that the indirect path for anxiety suggests that those who were satisfied with the Government’s handling were less prone to comply with recommendations since satisfaction was related to less anxiety and anxiety in turn was related to increased compliance. Hence, for both pride and anxiety, satisfaction with the handling essentially was associated to less compliance.

The only positive effect of satisfaction was that it also was related to higher compliance via compassion – those who felt compassion when thinking about the Swedish approach were more prone to follow the recommendations. This seems intuitive given that earlier research has found that empathy increased health promoting behaviors in relation to Covid-19. For instance, empathy increased physical distancing and wearing of face masks (Pfattheichera et al., 2020). There is a broad consensus in the literature on compassion that it involves feelings for others who are suffering and thus motivates action to help them (Goetz et al., 2010; Lazarus, 1991; Strauss et al., 2016). In the case of Covid-19, one social group in particular has been targeted as beneficiaries of others’ compliance with restrictions, namely the elderly. Covid-19 has proved especially dangerous for the elderly population, a group which already is seen as vulnerable and helpless. Hence, the experience of compassion may be particularly related to the desire to protect the elderly. In the Swedish news media and communication, the elderly was repeatedly pointed to as a vulnerable group in need of protection.

When it comes to anxiety, which is probably the emotion of these three that has received most empirical attention, it is an emotion that is elicited when a stimulus is ambiguous, uncertain, or unspecified. This could lead to a preference for increased security (Bäck et al., 2020), and anxiety has also been related to action (Soomro, 2012; Renström & Bäck, 2021). In the empirical literature, fear and anxiety are often grouped together, but some research indicate that these should be considered separate (Öhman & Mineka, 2001; Sylvers Lilienfeld & LaPrairie, 2011; Renström & Bäck, 2021). The present results further add to that, since there was no effect of fear on compliance. In fact, fear has been related to inaction (Renström & Bäck, 2021), and a meta-analysis of fear appeals (messages that arouse fear) show that fear sometimes leads to reactance or avoidance (Witte & Allen, 2000). In an earlier study of health behaviors in relation to Covid-19, Bigot et al., (2021) found that current state of fear/anxiety and health anxiety increased hand washing but had no effect on social distancing – indicating that emotions may have differential impact on different health promoting behaviors. In the current study, we have chosen to investigate compliance with recommendations in general and hence not engaged in analyses of separate health promoting behaviors. A compromising aspect of that study is that the authors grouped anxiety and fear together, which makes it difficult to compare to the results found in the present research. It is, further, important to remember here that fear is not fear of the virus, or becoming sick, it is fear elicited by the Swedish approach to handling Covid-19.

Interestingly, pride was associated with a lower tendency to follow the recommendations. Pride is an often neglected emotion and therefore the literature on its cognitive and behavioral consequences is scarce. Pride is often elicited by own or related other’s achievement and is associated with self-esteem, and positive self-image (Lea & Webley, 1997). Pride has also been shown to sometimes lead to irrational decisions (Lea & Webley, 1997) and antisocial behavior (Wubben, De Cremer & van Dijk, 2012). The literature makes a distinction between authentic or proper pride (i.e. confidence, accomplishment) and hubristic or false pride (vanity, arrogance) – where the former leads to prosociality but the latter leads to antisociality. It can hence be argued that the pride displayed among the participants in this study was of the latter kind since it was unrelated to prosocial behavior. A possible explanation could be found if we consider the current political climate at the time of the survey – since the spread of the virus was at an all-time low, everything indicated that the Swedish approach had succeeded, possibly leading the supporters of the approach to the conclusion that they were right and that the opponents were wrong. If this was the origin of the experience of pride, it would make sense that this would in fact be hubristic pride rather than authentic pride. Moreover, in an earlier study, Bigot et al (2021) found that a dimension of emotions, which they labelled enthusiastic/happy that also included the item proud, indeed led to less compliance in limiting social contacts.

The two positive emotions that significantly mediated the effect on health behaviors, pride and compassion, had opposite effects. At first glance, this may seem counterintuitive, but it seems that similarly to that negative emotions cannot be grouped together, so cannot positive emotions. Compassion followed the pattern that could be expected in this context – related to increased health behaviors while pride did not. As we have argued above, we believe that this may have to do with that pride in this situation could be hubristic pride rather than authentic and as such it could lead to negative outcomes.

Another interesting result was that shame mediated the effect of satisfaction with the Government’s handling on the tendency to wear face masks. People who were dissatisfied with the Government’s handling felt more ashamed, which predicted the use of face masks. Because, the Public Health Agency actually recommended against the use of face masks, an open display of wearing a face mask could be seen as a political statement – the display of dissatisfaction with the handling. The face mask hence, became a politicized symbol in the discussion about the Swedish approach to handling the outbreak of Covid-19. In support of that interpretation is that at the time of the study, spread of the virus was at an all-time low, many citizens’ summer holidays were still going on and hence there was not much crowding at for example public transportations. This indicates that the need of face masks in public to actually limit the spread was minimal. Shame is an emotion that is usually experienced when an individual has transgressed some social norm. Applying this to the current setting, the participants who experienced shame rather did it on behalf of the Swedish nation and Swedish Government, than feeling shameful about their own behavior. Hence, one way to show disapproval with this approach is to wear a face mask as a protest symbol. This is a stark contrast to how shame applied to the own behavior usually shows, where denial and fleeing behavior is more common. However, some research show that when individuals feel ashamed on behalf of their ingroup, this can motivate intentions to change ingroup self and behavior (Gausel & Brown, 2012).

Finally, there were no effects of anger, fear, or sadness, on either compliance nor wearing face masks. Even though people experience these emotions when thinking of the Swedish approach, it does not make them more or less inclined to engage in behaviors to stop the spread of the virus. The literature on fear appeals show that a fear appeal initiates two appraisals. First, individuals appraise the threat of an issue from a message. If this appraisal leads the individual to believe that they are susceptible to a serious threat, then they are motivated to begin the second appraisal, which evaluates the efficacy of the recommended response. If the threat is perceived as irrelevant or insignificant, then there is no motivation to engage in further processing and no action will result (Witte & Allen, 2000). It is important to remember here that participants responded to fear that they experienced when thinking about the Swedish approach to handling the Covid-19 outbreak and not to the recommendations, so it differs from a fear appeal in the traditional meaning of the concept. Nonetheless, it is plausible that they were thinking about the virus and experienced fear in relation to contracting the virus. However, this might have led only to a first appraisal. As was known by this time, the virus was not as dangerous as had been suggested earlier, and the appraisal process might have been aborted before entering the second stage, which is the stage necessary for action. However, it could also be that appraisals do enter the second stage, but that participants focused on controlling their fear instead of the threat, which also leads to inaction through denial, reactance or avoidance (Witte & Allen, 2000).

One interpretation of the lack of effects for many of the prominent emotions such as fear and anger, is that the polarization in the Swedish society regarding the handling of the Covid-19 situation seems to be mainly an attitudinal polarization related to the politics of the handling, and that the handling of the issue has become politicized. That does not mean that people will not engage in health promoting behaviors, which is a positive interpretation of the results – regardless of political attitudes, people may still engage in behaviors that limit the spread of the virus.

### Limitations and future directions

A few limitations are worth mentioning. First, we have measured compliance with the national recommendations and one behavior that was not recommended by the Public Health Agency. However, it is difficult to disentangle who complies with health promotion behaviors due to them being recommended by the Public Health Agency, or simply because they will limit the spread of the virus. However, and importantly, compliance does not equate satisfaction with the recommendations and handling. We only had one item measuring a health promoting behavior that was openly not endorsed by the Public Health Agency – the face mask. Two things compromise the findings of this item. First, it would be beneficial to include other behaviors that were not, at least not explicitly, recommended by the Public Health Agency, to achieve more generalizable results. Second, this survey was conducted in August–September, 2020, when the pandemic was at an all-time low and the use of face masks in public was frowned upon. It is therefore important that the results are interpreted in light of the context at the time. In the late fall/early winter 2020 the spread rapidly increased to an all-time high, and public wearing of face masks became more frequent despite the Public Health Agency’s consistent recommendations against it. Finally, the Public Health Agency changed their mind and recommended face masks in public transportation or crowded places. Both the fact that the spread increased, implying that the Swedish approach in fact was not successful but the low of the summer was rather a seasonal fluctuation in the spread, and the fact that the Public Health Agency wavered on the face mask issue, likely had a significant impact on how the Public Health Agency was viewed later on in the pandemic. It would be desirable to run a follow-up study to explore if the satisfaction of the handling of the Covid-19 outbreak has changed and if it has had any influence on health promoting behaviors. Also, it would be interesting to explore if the motivation behind the increased use of face masks was a political statement of disapproval or an increased sense of protection, or a combination of the two.

The results of the present research were fairly weak, even though the sample was large. Hence, other variables than emotions should be explored as well if the explanation of actual behavior is in focus. Yet, the effects of satisfaction with the Government’s handling on emotions were fairly strong, indicating that politics do elicit emotions, but how they affect behavior may vary, at least in this particular instance.

One alternative factor that future research may want to explore is the role of motivation. For instance, whether the motivation to adhere is internal or external as described by self-determination theory (Ryan & Deci, 2017). Another factor, which is also connected to emotional reactions, is risk-perception (Lerner et al., 2003). That is, the emotions elicited by thinking about the Swedish governments handling, could influence to what extent Covid-19 is seen as a real risk, which could then affect subsequent behavior. When explaining health behaviors, risk perceptions are often discussed in terms of more rational evaluations of the likelihood of harm if no action is taken and the severity of harm if no action is taken (Brewer et al., 2007). An interesting venue for future research is to explore how emotions interact with such perceptions when explaining health promoting behaviors during the Covid-19 pandemic. There are also individual-level factors that could be included, which could moderate the effect of how satisfied a person is with the Government’s handling and what emotions they experience. For instance, Right-wing authoritarianism is related to submission to authorities, but also to risk-perception (Altemeyer, 1998; Sibley & Duckitt 2008). Gender is another factor that could have influence given that emotions tend to be connected to gender stereotypes (Eagly, 1987; Plant et al., 2000).

Another relevant theoretical perspective to consider is how emotions evolve during a narrative, and how such changes may direct individuals to attend differently to different pieces of information (Nabi & Green, 2015). This idea of changes in emotions may be more relevant to consider in a larger perspective on the communication surrounding the Covid-19 pandemic over time, than in relation to the present article.

## Conclusions

An important conclusion of the present study is that emotions are important to political life and to individuals’ health promoting behaviors. Clearly, individuals react with a host of emotions when thinking about how Sweden handled the situation with the outbreak of the Covid-19 virus, and such emotions may have important consequences for how people view each other (see e.g. Iyengar et al., 2019; Renström et al., 2020). Even though emotions are an important aspect to consider in human behavior it is often neglected when governments and authorities design and present their recommendations. The present results highlight the importance of considering emotions in these situations.

## Footnotes

We chose the recommendations that were aimed at everybody and that influenced everyday life, which were most salient at the time of the study. There were other recommendations for people who were sick relating to testing and quarantine. We did not include them here since we believe that these may be influenced by different mechanisms.
